# The effect of peer support on fear of disease progression in patients with glioma: a protocol for systematic review and meta-analysis

**DOI:** 10.3389/fmed.2026.1864454

**Published:** 2026-07-02

**Authors:** Dandan Liang, Ruiying Jia, Qin Huang, Yanhong Pan

**Affiliations:** Department of Nursing, Second Affiliated Hospital, School of Medicine, Zhejiang University, Hangzhou, China

**Keywords:** fear of disease progression, glioma, meta-analysis, peer support, protocol, systematic review

## Abstract

**Background:**

Due to the characteristics of high recurrence rate, high disability rate and high mortality rate, glioma patients will have a high level of fear of disease progression (FoP), which further affects the happiness and quality of life of patients. Peer support is a powerful, sustained and affordable intervention that plays a positive role in many diseases by providing patients with the emotional, social and practical assistance necessary to manage their disease and maintain their health through a variety of intervention forms. Therefore, peer support may be an effective way to reduce FoP and improve mental health in patients with glioma.

**Methods:**

Eight databases (PubMed, Embase, Cochrane Library, CNKI, PsycNET, MEDLINE, Psychology & Behavioral Sciences Collection and Web of Science) will be used to select eligible studies that were published from inception to March, 2026. The eligible studies will be screened, extracted and then the methodological quality will be evaluated independently by two reviewers. RevMan version 5.3 software and Stata version 18.0 software will be used for meta-analysis.

**Conclusion:**

The results of this meta-analysis will provide evidence for the effectiveness of peer support in patients with glioma. In the future, this study will provide theoretical basis for developing individualized peer support intervention programs to improve adverse health emotions such as FoP, fatigue, depression and anxiety in glioma patients.

**Systematic review registration:**

PROSPERO CRD 42024556385.

## 1 Introduction

Glioma is a brain tumor that develops from an abnormal glial cell, the four subtypes of glioma are: Astrocytoma; Ependymoma; Glioblastoma and Oligodendroglioma, together they account for more than 78% of all primary brain tumors (1). Age is a known risk factor for glioma, but the biological basis of this association has not been elucidated, the external risk factor is exposure to ionizing radiation, and others include genetics and family history (2). In addition, the symptoms of glioma vary according to the location and size of the tumor, including headache, nausea, vomiting, memory problems, fatigue, and cognitive impairment. When some specific sites are involved, numbness, aphasia, loss of peripheral vision, and severe complications such as brain hemorrhage, brain herniation, hydrocephalus, and seizures may occur (1), causing great suffering to patients. At present, the treatment of glioma mainly includes surgery, radiotherapy, chemotherapy, immunotherapy, Laser interstitial thermal therapy (LITT), etc. (3–7), but the recurrence rate after treatment is high and the survival cycle is short. Studies have shown that the average survival time of patients with glioma is 20.04 months, the postoperative recurrence rate is 52.5%, the postoperative three-year survival rate is 17.5%, and the five-year survival rate is 2.5% (8). As a result, patients will have anxiety, depression and other adverse health psychological problems (9), among which FoP is one of the most common psychological reactions of cancer patients, and also one of the social psychological needs that cancer survivors cannot receive effective help for (10).

Dankert et al. (11) proposed FoP as an individual’s fear of everything related to his or her real-life disease. Specifically, FoP is defined as the various biopsychosocial consequences of the fear of disease progression or the fear of disease recurrence. It is reported that 33 to 96% of cancer patients worldwide have FoP, and the proportion with severe fear reaches 87% (12). A meta-analysis showed that the incidence of FoP among cancer survivors in China was 51.0%. Age, mean monthly income, clinical stage, educational level, marital status, employment status, course of disease, family history and anxiety were the main influencing factors for FoP among cancer survivors in China (13). Other studies have shown that high levels of FoP in cancer patients are positively correlated with higher levels of anxiety, depression, insomnia, pain, fatigue, and dysfunctional self-focus (14), and that controlling fear of disease progression may be an important factor in increasing hope and improving quality of life for cancer patients (15). Therefore, glioma, as a malignant tumor with high recurrence rate, high mortality rate and high disability rate, needs an effective intervention method to improve its FoP level.

Peer support refers to a variety of forms that enable people with similar diseases, physical conditions, or experiences to provide substantive help and emotional support to each other (16), building non-hierarchical reciprocal relationships by sharing similar life experiences with others, the more homogeneous peers, the more likely this support is to lead to understanding, empathy, and mutual help (17). At present, there are seven successful models of peer support intervention including professional-led group visits with peer exchange; peer-led face-to-face self-management programs; peer coaches; community health workers; support groups; telephone-based peer support; web- and e-mail-based programs (18). In addition, the core functions of peer support include: (1) assistance in daily management, (2) social and emotional support, (3) linkage to clinical care and community resources, and (4) ongoing availability of support (19). By providing technical, experiential and emotional support, peer supporters can increase patients’ hope and inspiration, enhance their self-esteem and confidence, and improve engagement in self-care and wellness, reducing their psychoneurotic symptoms such as depression (20), and thus improving their self-efficacy and FoP levels, not only provides benefits for the patients themselves, but also reduces the burden on their families.

At present, there have been some studies on the effects of peer support on mental health problems in people with type 2 diabetes, heart disease, breast cancer, obesity, etc. (21–24). However, there is no meta-analysis or systematic review on peer support intervention to improve FoP in patients with glioma. Therefore, this study builds upon our previous work on remote peer support in patients with breast cancer during the COVID-19 pandemic (25), but extends it to a new population (glioma patients) and a different outcome (fear of disease progression). In order to find an effective way to improve FoP in glioma patients, this study conducted a meta-analysis of randomized controlled trials on the improvement of FoP in patients with glioma based on peer support intervention.

## 2 Methods and analysis

This systematic review has been registered in PROSPERO CRD 42024556385. We strictly abide by the Preferred Reporting Items for Systematic Review and Meta-Analysis Protocols (PRISMA-P) guidelines (26).

### 2.1 Eligibility criteria

Eligibility criteria follows the PICOS framework regarding population, intervention, comparator, outcome, and study type.

### 2.2 Population

Patients diagnosed with glioma (Grade I, II, III, and IV glioma patients) will be included, regardless of age, gender, educational status or racial restrictions. People with other serious physical diseases (such as stroke, myocardial infarction, or other malignant tumors) will be excluded.

### 2.3 Interventions

The intervention group adopted peer support or the combination of peer support and usual care. Peer supporters were people with glioma or people caring for someone with glioma, but not health professionals.

### 2.4 Comparator

The comparator group included participants who accepted usual care, usual education, etc.

### 2.5 Outcomes

The primary outcomes of the study are FoP, the secondary outcome will be fatigue, depression and anxiety.

### 2.6 Studies

This study will include randomized controlled trials investigating the effect of peer support on patients with glioma. Duplicate research reports or insufficient data will be excluded.

### 2.7 Language

Published in English or Chinese.

### 2.8 Search strategy

The following electronic databases were utilized for selecting eligible studies published from inception to March, 2026: PubMed, Embase, Cochrane Library, CNKI, PsycNET, MEDLINE, Psychology & Behavioral Sciences Collection and Web of Science. The search strategy in PubMed is as follows:

- #1 Search: “Glioma”[Mesh] Sort by: Most Recent
- #2 Search: ((Gliomas[Title/Abstract]) OR (Glial Cell Tumors[Title/Abstract])) OR (Glial Cell Tumor[Title/Abstract])) OR (Tumor, Glial Cell[Title/Abstract])) OR (Tumors, GlialSearch: (Cell[Title/Abstract])) OR (Mixed Glioma[Title/Abstract])) OR (Glioma, Mixed[Title/Abstract])) OR (Gliomas, Mixed[Title/Abstract])) OR (Mixed Gliomas[Title/Abstract])) OR (Malignant Glioma[Title/Abstract])) OR (Glioma, Malignant[Title/Abstract])) OR (Gliomas, Malignant[Title/Abstract])) OR (Malignant Gliomas[Title/Abstract]) Sort by: Publication Date
- #3 Search: #1 OR #2
- #4 Search: (((((((peer support[Title/Abstract]) OR (peer education[Title/Abstract])) OR (peer-led[Title/Abstract])) OR (peer coach[Title/Abstract])) OR (peer counsel[Title/Abstract])) OR (peer mentor[Title/Abstract])) OR (peer group[Title/Abstract])) OR (peer health[Title/Abstract]) Sort by: Publication Date
- #5 Search: ((randomized controlled trial[Title/Abstract]) OR (controlled clinical trials, randomized[Title/Abstract])) OR (RCT[Title/Abstract]) Sort by: Publication Date
- #6 Search: #3 AND #4 AND #5

### 2.9 Study selection

**Figure 1:**
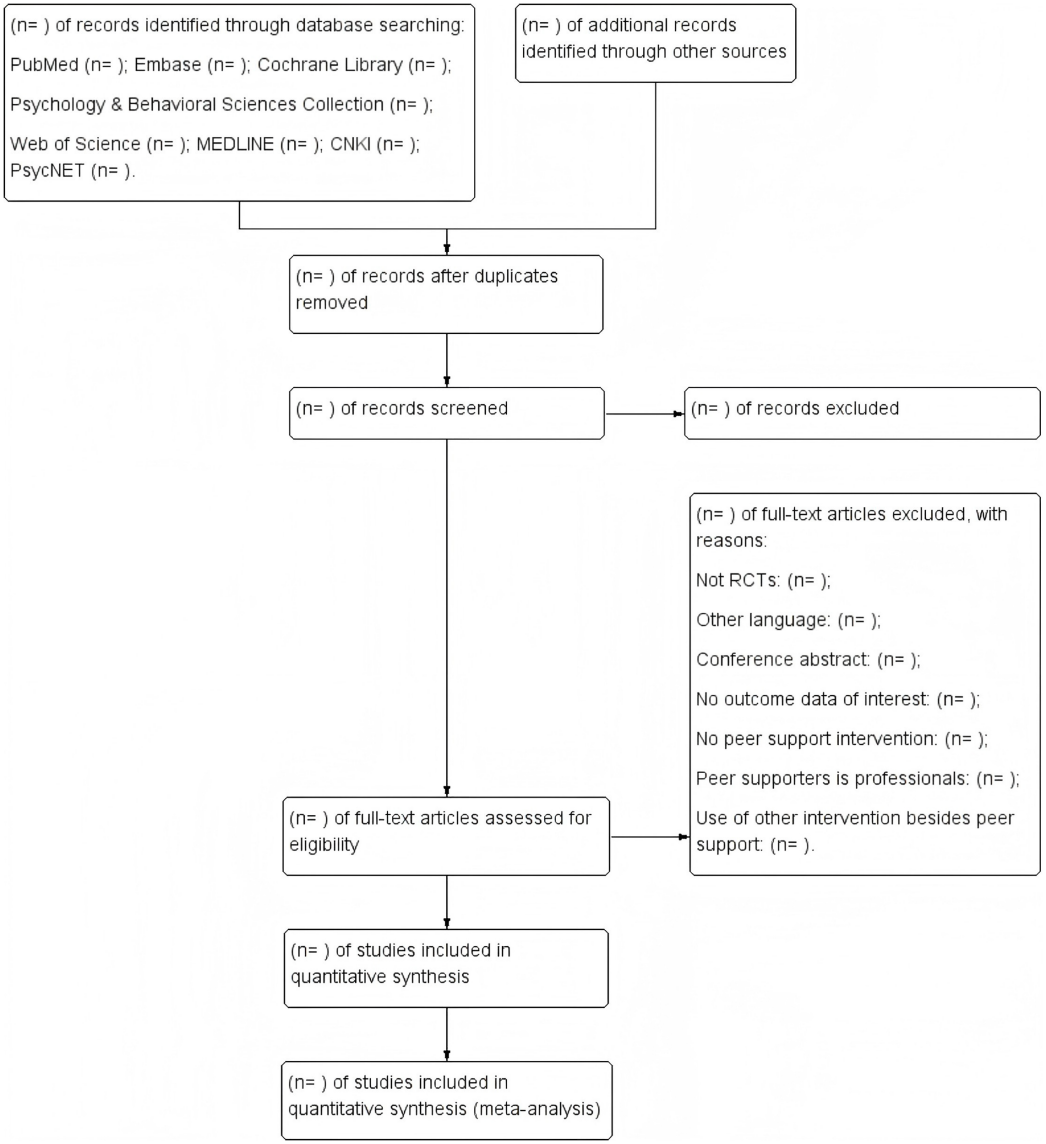
Flow diagram of the literature screening process and results. This figure is reused from our previous publication (25).

Relevant abstracts and titles for all studies will be screened and evaluated by 2 independent reviewers against predefined inclusion criteria, then duplicates or ineligible articles will be excluded based on relevant reasons. The third investigator will resolve any disagreement between the 2 reviewers. The process of screening selection is shown in Figure 1.

### 2.10 Data collection and management

Two reviewers will independently collect data from all studies based on the data extraction form. The following study information will be recorded: first author, year of publication, country, sample size, participant age, tumor location and grade, recruitment site, treatment methods, peer support intervention duration and mode, outcome measures, specific treatment for the control groups, follow-up time and intervention content. The third investigator will resolve any disagreement between the two reviewers. The authors were contacted to obtain missing or unclear data for further analyses. If it fails, we will analyze it based on available data.

### 2.11 Subgroup analysis

To explore potential sources of clinical and methodological heterogeneity, we will conduct subgroup analyses if data are available for the following factors: intervention mode, intervention duration, age of participant, country, tumor location and grade, treatment method, and outcome measures.

### 2.12 Assessment of risk of bias

We will use the tool recommended by the Cochrane Handbook Version 5.1.0 (the Cochrane risk-of-bias tool) (27) to analyze the risk of bias in the trials from the following seven aspects: random sequence generation, allocation concealment, blinding of participants and personnel, blinding of outcome assessment, incomplete outcome data, selective reporting, and other bias. Every item will be classified as “Yes” (low risk of bias), “No” (high risk of bias), or “Unclear” (moderate risk of bias). When the risk of bias of all seven components were defined as “low risk of bias,” the trial was defined as the overall “low risk of bias.” At the same time, when one or more of the seven bias components were classified as high risk, the trial was graded as “High risk of bias.” In other cases, the trial was graded as “Unclear risk.” Disagreements in bias classification were resolved by discussions among the two reviewers and, if necessary, through discussions with the authors.

### 2.13 Data synthesis and analysis

We will enter group means, standard deviations (SD), and the number of participants in RevMan 5.3 and conducted a random-effects model meta-analysis, and Stata18.0 will be used for sensitivity analysis and Egger’s test. If the evaluated trials used different scales to measure the same outcomes, data will be synthesized by using Hedge’s g of standardized mean difference (SMD) with 95% confidence interval (CI). According to the Cochrane Handbook, Chi-Squared test and *I*^2^ value could be used to evaluate the heterogeneity. *I*^2^ values of 25, 50, and 75% are considered as low, moderate, and high heterogeneity, respectively. We will use sensitivity analysis to examine the stability of the results by removing individual trials to determine whether the removed study had a particular effect. Furthermore, after a sensitivity analysis was performed while excluding studies with a high risk of bias, we will perform funnel plots and visually examine the signs of asymmetry to investigate publication bias, then use Egger’s test (28) as a formal test of publication bias when the number of the included studies was more than 10 (*n* ≥ 10).

### 2.14 Ethics and dissemination

This work does not require relevant ethical review because there is no data linked to individual patient or animal information. Our research results will be shared and demonstrated through peer-reviewed journals.

## 3 Discussion

With the development of society and the progress of medicine, more and more treatment methods for glioma are being explored (4, 5). However, due to the characteristics of high recurrence rate, high disability rate and high mortality rate, glioma patients will have a high level of FoP, which further affects the happiness and quality of life of patients. Peer support is a powerful, sustained and affordable intervention that plays a positive role in many diseases by providing patients with the emotional, social and practical assistance necessary to manage their disease and maintain their health through a variety of intervention forms (20). Research has shown that peer support can improve anxiety and depression in cancer patients, enhance their quality of life, greatly benefit cancer patients, and can be used as a supplement to traditional health care services during cancer recovery (29). However, there is still no meta-analysis on the effectiveness of peer support on FoP in patients with glioma. In this paper, we will systematically evaluate whether peer support has a positive effect on FoP in patients with glioma.

We hope that this study will provide evidence for peer support to reduce FoP and improve mental health in patients with glioma. In addition, personalized interventions can be developed according to the physical and mental characteristics of patients with glioma to maximize the role of peer support.

## 4 Conclusion

The purpose of this study was to evaluate the effect of peer support on FoP, fatigue, depression and anxiety in patients with glioma. The results may offer hope for reducing mental health problems and improving quality of life in patients with glioma.
